# Coronary artery calcification on low-dose chest CT is an early predictor of severe progression of COVID-19—A multi-center, multi-vendor study

**DOI:** 10.1371/journal.pone.0255045

**Published:** 2021-07-21

**Authors:** Philipp Fervers, Jonathan Kottlors, Nils Große Hokamp, Johannes Bremm, David Maintz, Stephanie Tritt, Orkhan Safarov, Thorsten Persigehl, Nils Vollmar, Paul Martin Bansmann, Nuran Abdullayev

**Affiliations:** 1 Department of Radiology, University Hospital of Cologne, Cologne, Germany; 2 Department of Radiology, Helios Dr. Horst Schmidt Kliniken Wiesbaden, Wiesbaden, Germany; 3 Department of Radiology, Krankenhaus Porz am Rhein, Cologne, Germany; University "Magna Graecia" of Catanzaro, ITALY

## Abstract

**Purpose:**

Cardiovascular comorbidity anticipates severe progression of COVID-19 and becomes evident by coronary artery calcification (CAC) on low-dose chest computed tomography (LDCT). The purpose of this study was to predict a patient’s obligation of intensive care treatment by evaluating the coronary calcium burden on the initial diagnostic LDCT.

**Methods:**

Eighty-nine consecutive patients with parallel LDCT and positive RT-PCR for SARS-CoV-2 were included from three centers. The primary endpoint was admission to ICU, tracheal intubation, or death in the 22-day follow-up period. CAC burden was represented by the Agatston score. Multivariate logistic regression was modeled for prediction of the primary endpoint by the independent variables “Agatston score > 0”, as well as the CT lung involvement score, patient sex, age, clinical predictors of severe COVID-19 progression (history of hypertension, diabetes, prior cardiovascular event, active smoking, or hyperlipidemia), and laboratory parameters (creatinine, C-reactive protein, leucocyte, as well as thrombocyte counts, relative lymphocyte count, d-dimer, and lactate dehydrogenase levels).

**Results:**

After excluding multicollinearity, “Agatston score >0” was an independent regressor within multivariate analysis for prediction of the primary endpoint (p<0.01). Further independent regressors were creatinine (p = 0.02) and leucocyte count (p = 0.04). The Agatston score was significantly higher for COVID-19 cases which completed the primary endpoint (64.2 [interquartile range 1.7–409.4] vs. 0 [interquartile range 0–0]).

**Conclusion:**

CAC scoring on LDCT might help to predict future obligation of intensive care treatment at the day of patient admission to the hospital.

## Introduction

Coronavirus Disease 2019 (COVID-19) most commonly manifests by unspecific and mild symptoms [1]. Severe complications include respiratory failure, sepsis and acute cardiac injury, which regularly imply obligation of intensive care treatment and invasive ventilation [2–5]. Such severe progression of COVID-19 is promoted by preexisting comorbidity and, above all, cardiovascular risk factors (CRF) [2, 3, 5–8]. I.e., the most commonly reported risk factors for severe complications of COVID-19 include arterial hypertension, diabetes and history of a cardiovascular event [8].

One aspect of cardiovascular disease is atherosclerotic transformation of the vessel wall. Vessel calcification, such as coronary artery calcification (CAC), represent the lifetime cumulative burden of all risk factors for atherosclerotic artery disease [9–11]. Generally acknowledged risk factors for development of atherosclerotic calcification include high age, active smoking, diabetes mellitus, dyslipidemia and arterial hypertension [12]. These promoters of atherosclerosis are also known to entail a poor prognosis in COVID-19, as explained above.

Low-dose chest CT (LDCT) is regularly performed in the context of COVID-19 to recognize complications, to confirm the diagnosis of laboratory testing, and for staging of the disease [13]. Aside from evaluation of the lungs, LDCT allows for assessment of the heart and coronary vessels to a limited extent. Calcified atherosclerotic plaques stand out by the high intrinsic contrast to the adjacent soft tissue. The most commonly used and best evaluated grading for clinical quantification of CAC is the Agatston score [10]. The Agatston score respects overall volume and maximum attenuation of calcified atherosclerotic plaques on a CT scan. Several studies demonstrated comparable CAC-scoring results of ECG-gated, dedicated calcium scoring CTs and ungated LDCTs, which implies the feasibility to estimate the Agatston score based on a COVID-19 LDCT [14, 15].

Hence, the hypothesis of this study is that the Agatston score obtained from LDCT can serve as a surrogate for cardiovascular comorbidity to identify patients at risk of severe COVID-19 progression.

## Methods

All procedures performed in studies involving human participants were in accordance with the ethical standards of the institutional and national research committee and with the 1964 Helsinki declaration and its later amendments. Informed consent was waived due to retrospective study characteristics. The study was approved by the local ethics committee of the corresponding author’s institution (ethics committee of the Medical Faculty of the University Cologne, application number 20–1219). All imaging was performed for clinical indications. No scan was conducted explicitly for the purpose of this study.

### Patient enrollment

Study inclusion was based on screening of the picture archiving and communication system (PACS) and electronic medical records at three level-I hospitals (XX, XX and XX). Diagnostic imaging was indicated by clinical considerations. At each institution LDCT was performed for patients with suspected or RT-PCR positive COVID-19 and

1. moderate to severe clinical features, or
2. worsening of respiratory status.

Inclusion criteria to our study comprised

1. LDCT at initial presentation at hospital according to the hereafter specified imaging protocol,
2. imaging between 10^th^ of March and 30^th^ of June, 2020, as well as imaging between 30^th^ of June and 15^th^ of October, 2020 at center 1,
3. mouth swab positive for SARS-CoV-2 as indicated by RT-PCR, and
4. age ≥18 years.

One patient was excluded due to extensive beam hardening artifacts, impeding the identification of CAC. Twelve patients were excluded due to history of coronary artery disease. This resulted in a total of 89 patients (61, 14, and 14 at centers 1, 2, and 3, respectively).

### Clinical follow-up, comorbidity, and laboratory tests

A radiologist reviewed the clinical records for the primary endpoint “admission to ICU, tracheal intubation or death” at each site. Follow-up time was 22 days after the initial LDCT, based on reported data [6]. Clinical outcome was dummy encoded for each patient by completion of the primary endpoint events (0/1). Comorbidities were reviewed from the clinical records and dummy encoded (0/1) for each of the five following items: 1. history of arterial hypertension or current anti-hypertensive medication, 2. history of diabetes or current anti-diabetic medication, 3. prior cardiovascular event (myocardial infarction or revascularization procedure, stroke or transient ischemic attack, or diagnosis of peripheral arterial disease), 4. history of hyperlipidemia or lipid-lowering agents in current medication, 5. active smoking habit. Further, commonly reported laboratory values for high-risk COVID-19 were noted from the clinical records (creatinine, C-reactive protein, leucocyte as well as thrombocyte counts, relative lymphocyte count, d-dimer, and lactate dehydrogenase levels) [16–19]. Laboratory tests were performed on the day of the CT scan (+/- 1 day).

### LDCT scanning protocol and image reconstruction

All scans were performed on clinically available CT scanners (iCT 256, Philips Healthcare, SOMATOM Definition AS+, Siemens Healthineers, and Revolution ACT, GE Healthcare). Patients were placed in a head-first supine position. No contrast agent was administered.

Scan parameters for the Philips scanner were: collimation 80.0 × 0.6 mm, pitch 0.76, tube voltage 120 kV, mean CTDIvol 2.1 ± 0.6 mGy, mean DLP 87.4 ± 28.3 mGy*cm (Siemens: collimation 38.4 × 0.6 mm, pitch 1.2, tube voltage 120 kV, CTDIvol 6.8 ± 2.6 mGy, mean DLP 232.4 ± 95.0 mGy*cm; GE: collimation 20.0 × 1.3 mm, pitch 1.38, tube voltage 120 kV, CTDIvol 5.0 ± 3.2 mGy, mean DLP 172.4 ± 97.3 mGy*cm).

For the Philips scanner, images were reconstructed in a 512 x 512 matrix containing pixels of 0.68 x 0.68 mm. Slice thickness was 2.0 mm with an overlap of 1.0 mm (Siemens: 512 x 512 pixels of 0.64 x 0.64 mm, slice thickness 1.0 mm, overlap 0 mm; GE: 512 x 512 pixels of 0.70 x 0.70 mm, slice thickness 1.3 mm, overlap 0.6 mm).

### Calcium quantification and lung involvement score

**Fig 1:**
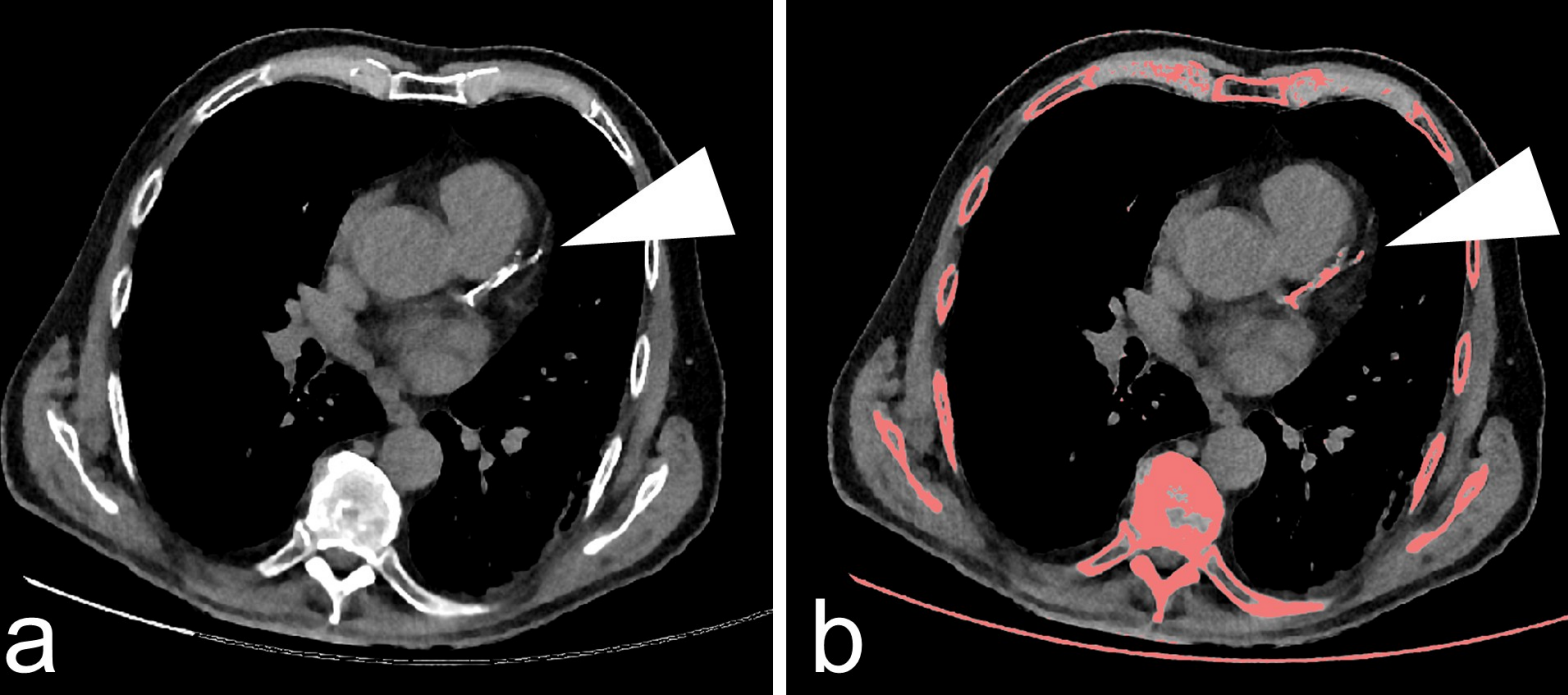
Quantification of coronary artery calcification. Exemplary axial computed tomography slice of a patient with evidence of coronary artery calcification (a). Connected voxels with an attenuation above 130 HU and minimum volume of 0.5 mm^3^ were highlighted by the vendor’s software (b). Coronary artery plaques were manually selected, excluding aortic valve calcifications, e.g., calcification of the *Ramus interventricularis anterior* (white arrowheads). The Agatston score was noted for each patient.

Assessment of the Agatston score was performed using the vendor’s software (IntelliSpace Portal, HeartBeat-CS, Philips Healthcare). The software highlighted all regions with a minimum volume of 0.5 mm^3^ and attenuation above 130 HU with a colored overlay (Fig 1). A blinded observer manually identified highlighted lesions with a mouse click. CAC clearly associated with aortic valve calcifications were excluded from the measurements. The software automatically performed three-dimensional volumetry of all connected voxels and calculated the Agatston score without specific user interaction.

Lung involvement was assessed semi-quantitatively for each patient, using the COVID-19 chest CT score established by Francone et al. [20]. Based on the extent of lobar involvement in COVID-19 pneumonia, each lobe is addressed by a particular score from 0–5 (0: 0%; 1: < 5%; 2: 5–25%; 3: 26–50%; 4: 51–75%; 5: > 75%). The total score is obtained by the sum of lobar scores (global lung involvement score with range 0–25).

### Statistics and data analysis

Data analysis was carried out in *R* language for statistical computing (R Foundation, Vienna, Austria, version 4.0.0). Wilcoxon test was performed for comparison of ordinal data and non-parametric data, chi-squared test for nominal data, and student’s t-test for parametric data. Shapiro-Wilk’s test was performed to test data for normality, using the *R* library *dplyr* [21]. Multivariate regression was implemented by the *glm()* function in “binomial” mode. As a measure for the accuracy of fit of the logistic regression, pseudo-R^2^ was calculated by the Nagelkerke method (> 0.2 acceptable, > 0.4 good, > 0.5 very good) [22]. The variance inflation factor (VIF) was calculated for each independent variable using the *cars* package for *R* to preclude multicollinearity (< 2.5 no significant multicollinearity) [23, 24]. Statistical power was calculated post-hoc using G*Power [25]. Intra- and inter-observer variability were reported on a randomly selected subset of 18% of our data by the intraclass correlation coefficient (ICC), using the *R* library *psych* [26, 27].

All mentioned values are stated as average ± standard deviation or median [first quartile, third quartile]. A p-value ≤0.05 was considered to indicate statistical significance.

## Results

Eighty-nine patients were evaluated (49 male, mean age 58.1 ± 15.9 years). The primary endpoint was observed in 36 patients (40.4%), seven patients deceased during the follow-up period (mortality rate 7.9%). All deceased patients died during hospitalization (three patients deceased in the ICU, four in an isolation unit). The most commonly reported cause of death was severe respiratory distress (five patients), followed by acute decompensated heart failure (two patients). Patient details are summarized in Table 1.

**Table 1:** Patient details.

	no primary endpoint in 22 days follow-up (n = 53)	ICU, tracheal intubation or death (n = 36)	Data available
Most severe clinical outcome in 22 days follow-up	No hospitalization (47.2%), hospitalization (52.8%)	Admission to ICU (36.2%), tracheal intubation (44.4%), death (19.4%)	100.0%
Agatston score***	0 [Q1 = 0, Q3 = 0]	64.2 [Q1 = 1.7, Q3 = 409.4]	100.0%
CT lung involvement score*	10.6 ± 5.1	12.8 ± 5.0	100.0%
Patient age***	52.0 ± 14.2	67.2 ± 14.0	100.0%
Patient sex	52.8% male	58.3% male	100.0%
Arterial hypertension	45.3%	61.1%	100.0%
Diabetes*	7.5%	30.1%	100.0%
Prior cardiovasc. event	11.3%	19.4%	100.0%
Hyperlipidemia	22.6%	27.8%	100.0%
Active smoker	9.4%	22.2%	100.0%
Creatinine** (norm: 0.5–1.1 mg/dl)	0.9 [Q1 = 0.7, Q3 = 1.1]	1.1 [Q1 = 0.8, Q3 = 1.7]	97.8%
C-reactive protein* (norm: <5.0 mg/l)	32.9 [Q1 = 9.0, Q3 = 71.8]	54.2 [Q1 = 25.9, Q3 = 97.5]	97.8%
Leucocyte count** (norm 4.4–11.3 x1E9/l)	6.6 [Q1 = 4.6, Q3 = 9.9]	9.6 [Q1 = 5.9. Q3 = 13.0]	97.8%
Thrombocyte count (norm: 150–400 x1E9/l)	186 [Q1 = 147, Q3 = 250]	201 [Q1 = 127, Q3 = 268]	97.8%
Rel. lymphocyte count* (norm: 19.1–47.9%)	23.5 [Q1 = 15.0, Q3 = 38.4]	14.5 [Q1 = 8.8, Q3 = 32.8]	94.4%
D-dimer** (norm: <0.5 μg/l)	0.7 [Q1 = 0.4, Q3 = 1.2]	1.7 [Q1 = 0.9, Q3 = 2.8]	73.0%
Lactate dehydrogenase (norm: < 245 U/l)	276.0 [Q1 = 223.0, Q3 = 373.5]	350.5 [Q1 = 249.5, Q3 = 478.3]	97.8%

*/**/*** differ significantly for COVID-19 cases with observed primary endpoint vs. no primary endpoint (Wilcoxon test, two-sided T-test, chi-squared test

*p<0.05

**p<0.01

***p<0.001)

A multivariate logistic regression model was fit to predict the primary endpoint by the main independent variable “Agatston score >0”, as well as the control variables patient age, sex, CT lung involvement score, and the five considered clinical anamnestic risk factors. To identify the most relevant laboratory parameters from the abundancy of available tests, only those parameters were respected in the regression model, which differed significantly between the two study groups. To acknowledge possible multicollinearity issues, the VIFs were calculated for each independent variable of our preliminary model. Independent variables with VIF >2.5 were dismissed from the model from top to bottom, ultimately removing the two clinical parameters hyperlipidemia (VIF = 6.6) and arterial hypertension (VIF = 2.8). The final regression model is summarized in Table 2.

**Table 2:** Multivariate logistic regression model for completion of the primary endpoint “admission to ICU, tracheal intubation or death” in COVID-19 during follow-up of 22 days.

Independent variable	p-value	Variance inflation factor	Odds ratio [95% confidence interval]
Agatston score >0	<0.01	2.4	33.6 [4.2–456.8]
CT lung involvement score	0.72	1.5	1.0 [0.8–1.3]
Patient age	0.56	2.2	1.0 [0.9–1.1]
Patient sex	0.18	1.4	0.3 [0.04–1.6]
Diabetes	0.52	1.6	0.4 [0.03–4.9]
Prior cardiovasc. event	0.06	1.9	0.1 [0.06–0.9]
Active smoker	0.06	2.2	19.7 [1.1–826.9]
Creatinine	0.02	1.8	10.2 [1.7–95.6]
C-reactive protein	0.56	1.3	1.0 [1.0–1.0]
Leucocyte count	0.04	1.9	1.3 [1.0–1.8]
Rel. lymphocyte count	0.27	1.8	1.0 [0.9–1.0]
D-dimer	0.83	1.4	1.0 [0.8–1.4]

After adjusting for the included control variables, the main independent variable “Agatston score >0” was significantly associated with the primary outcome within the multivariate logistic regression (p<0.01, odds ratio (OR) 33.6 [4.2–456.8]). Further, the creatinine level (p = 0.02, OR 10.2 [1.7–95.6]) and leucocyte count (p = 0.04, OR 1.3 [1.0–1.8]) were significant regressors of the primary outcome. Assuming average values for the control variables, patients with Agatston score 0 had a 0.16 risk (standard error 0.09) to complete the primary endpoint within 22 days. An Agatston score of >0 imposed to a risk of 0.86 (standard error 0.10) to complete the primary endpoint. Pseudo-R^2^ by Nagelkerke as an indicator for the accuracy of fit of the regression model was 0.64. That is, 64% of the variance of the dependent variable “primary endpoint observed” is accounted for by our regression, which is rated a “very good” model. As a comparison, a multivariate logistic regression model comprising only patient age, sex, the three considered clinical risk factors, and laboratory parameters achieved a pseudo-R^2^ of 0.50. Sensitivity and specificity to recognize completion of the primary endpoint by the dichotomous variable “Agatston score >0” within our study population was 0.75 (27/36) and 0.83 (44/53), respectively. The positive predictive value was 0.75 (27/36).

Statistical power for regression of the primary endpoint by the main independent variable in our multivariate model was >0.99, which exceeds the typically desired power level of 0.80 (S1 File). Intra- and inter-observer reliability for evaluation of Agatston scores and lung involvement were excellent (S2 File).

## Discussion

From the beginning of the COVID-19 pandemic Chinese physicians observed a coincidence of cardiovascular comorbidity and severe progression of the disease, which was consistently confirmed by international authors [2–6, 8]. However, a comprehensive clinical assessment of cardiovascular comorbidity might not be achievable for every patient during the pandemic, since it consumes time and medical resources. Instead, we suggest assessment of CAC on LDCT as a surrogate of individual cardiovascular comorbidity to anticipate severe progression of COVID-19. Coronary calcium scoring on LDCT has been proven viable in several studies [14, 15]. An experienced radiologist can obtain the Agatston score from a COVID-19 LDCT in a few seconds and automated software using deep learning is readily available [28]. In our study, the detection of CAC resulted in an increased risk to require intensive care treatment from 0.16 (standard error 0.09) to 0.86 (standard error 0.10). Our data suggest that the detection of CAC is an independent risk factor for severe progression of COVID-19, which remained significant after inclusion of demographic and clinical risk factors to the regression model (p<0.01, VIF = 2.4). The most accurate prediction of the primary endpoint was achieved by a model combining CAC, demographic and clinical risk factors (pseudo-R^2^ by Nagelkerke 0.64, “very good” model). This implies that a combination of calcium scoring on LDCT, basic laboratory tests, assessment of CT lung involvement, and a quick anamnesis for comorbidities explain 64% of the variance of the risk of ICU admission in the context of COVID-19.

Several recent studies explored the influence of CAC on the prognosis of COVID-19. An early single-center study from Italy identified a cutoff at Agatston score >400 as a risk factor for admission to an ICU or patient death on a population of 53 patients with subclinical coronary artery disease [29]. In a further single-center study by Dillinger et al., detection of CAC was independently associated with the primary outcome (mechanical noninvasive or invasive ventilation, extracorporeal membrane oxygenation, patient death) after inclusion of demographic and clinical risk factors (age, sex, hypertension, smoking, diabetes) [30]. The largest study up-to-date reports >1000 patients from Italy and approved CAC as an independent risk factor for in-hospital mortality [31]. However, to the best of our knowledge, this work is the first research to confirm CAC as an independent biomarker for severe COVID-19 after adjusting for demographic, anamnestic, and laboratory factors, as well as lung involvement in LDCT.

Limitations of our study include a small patient population, which leads to relatively wide 95% confidence intervals for our independent variables. Even though the association of our main independent variable to completion of the primary endpoint is highly significant (p<0.01), the wide OR confidence interval precludes accurate specification of the strength of the relationship. Further, endpoints of our statistical analysis were simplified to predict the clinical outcome that we recognized as most essential, considering the major issue of scarce intensive care capacities in the ongoing pandemic [32]. The reconstructed slice thickness of the CT scans in our study varied between 1 mm and 2 mm for the three centers, which should not affect measurements of CAC on non ECG-gated CT [33]. Similarly, modification of the tube current and consequently the radiation dose of a CT scan affects the standard deviation of measurements (noise), but not the average CT density [34]. An inter-vendor analysis to exclude a further scanner-specific bias was precluded by our sample size. Further, our work investigates only those individuals with COVID-19, who qualified for medical imaging due to clinical considerations (worsening of respiratory status, moderate to severe clinical features); any clinical application based on this study should be limited to such patient population. Future studies should be realized in a prospective setting and include a larger patient population to elaborate on the predictive benefit of CAC. A uniform study population concerning the days after symptom onset might help to investigate the predictive power of lung involvement, compared to the more stable anamnestic risk factors and the CAC burden.

During the ongoing COVID-19 pandemic, opportunistic quantification of CAC might allow for early recognition of patients at risk to require intensive care treatment. Our data indicate that evaluation of the CAC burden on LDCT can help to delimitate the number of required intensive care beds on the day of patient admission to the hospital. Since ICU capacity is a major bottleneck in the ongoing pandemic, this might facilitate resource management and collaboration on a national and international scale, ultimately promoting containment of COVID-19.

## Supporting information

S1 Data Patient details. (CSV)

S1 File Statistical power analysis in G*Power. (DOCX)

S2 File Intra- and inter-observer variability analysis. (DOCX)

## References

[1] Bansal M. Cardiovascular disease and COVID-19. Diabetes Metab Syndr Clin Res Rev. 2020;14: 247–250. doi: 10.1016/j.dsx.2020.03.013 32247212 PMC7102662

[2] Driggin E, Madhavan M V., Bikdeli B, Chuich T, Laracy J, Biondi-Zoccai G, et al. Cardiovascular Considerations for Patients, Health Care Workers, and Health Systems During the COVID-19 Pandemic. Journal of the American College of Cardiology. Elsevier USA; 2020. pp. 2352–2371. doi: 10.1016/j.jacc.2020.03.031 PMC7198856 32201335

[3] Guzik TJ, Mohiddin SA, Dimarco A, Patel V, Savvatis K, Marelli-Berg FM, et al. COVID-19 and the cardiovascular system: implications for risk assessment, diagnosis, and treatment options. Cardiovasc Res. 2020. doi: 10.1093/cvr/cvaa106 32352535 PMC7197627

[4] Du Y, Tu L, Zhu P, Mu M, Wang R, Yang P, et al. Clinical Features of 85 Fatal Cases of COVID-19 from Wuhan: A Retrospective Observational Study. SSRN Electron J. 2020. doi: 10.1164/rccm.202003-0543OC 32242738 PMC7258652

[5] Chen T, Wu D, Chen H, Yan W, Yang D, Chen G, et al. Clinical characteristics of 113 deceased patients with coronavirus disease 2019: retrospective study. BMJ. 2020;368: m1091. doi: 10.1136/bmj.m1091 32217556 PMC7190011

[6] Zhou F, Yu T, Du R, Fan G, Liu Y, Liu Z, et al. Clinical course and risk factors for mortality of adult inpatients with COVID-19 in Wuhan, China: a retrospective cohort study. Lancet. 2020;395: 1054–1062. doi: 10.1016/S0140-6736(20)30566-3 32171076 PMC7270627

[7] Li B, Yang J, Zhao F, Zhi L, Wang X, Liu L, et al. Prevalence and impact of cardiovascular metabolic diseases on COVID-19 in China. Clinical Research in Cardiology. Springer; 2020. pp. 531–538. doi: 10.1007/s00392-020-01626-9 PMC7087935 32161990

[8] Nishiga M, Wang DW, Han Y, Lewis DB, Wu JC. COVID-19 and cardiovascular disease: from basic mechanisms to clinical perspectives. Nature Reviews Cardiology. 2020. doi: 10.1038/s41569-020-0413-9 32690910 PMC7370876

[9] Berman DS, Arnson Y, Rozanski A. Coronary Artery Calcium Scanning: The Agatston Score and Beyond. JACC: Cardiovascular Imaging. Elsevier Inc.; 2016. pp. 1417–1419. doi: 10.1016/j.jcmg.2016.05.020 27931526

[10] Rusnak J, Behnes M, Henzler T, Reckord N, Vogler N, Meyer M, et al. Comparative analysis of high-sensitivity cardiac troponin i and T for their association with coronary computed tomography-assessed calcium scoring represented by the Agatston score. Eur J Med Res. 2017;22. doi: 10.1186/s40001-017-0263-z 29145895 PMC5691858

[11] Stary HC, Chandler AB, Dinsmore RE, Fuster V, Glagov S, Insull W, et al. A definition of advanced types of atherosclerotic lesions and a histological classification of atherosclerosis: A report from the Committee on Vascular Lesions of the council on arteriosclerosis, American heart association. Circulation. Lippincott Williams and Wilkins; 1995. pp. 1355–1374. doi: 10.1161/01.cir.92.5.1355 7648691

[12] Djekic D, Angerås O, Lappas G, Fagman E, Fagerberg B, Bergström G, et al. Impact of socioeconomic status on coronary artery calcification. Eur J Prev Cardiol. 2018;25: 1756–1764. doi: 10.1177/2047487318792103 30095278

[13] Kwee TC, Kwee RM. Chest ct in covid-19: What the radiologist needs to know. Radiographics. 2020;40: 1848–1865. doi: 10.1148/rg.2020200159 33095680 PMC7587296

[14] Budoff MJ, Nasir K, Kinney GL, Hokanson JE, Barr RG, Steiner R, et al. Coronary artery and thoracic calcium on noncontrast thoracic CT scans: Comparison of ungated and gated examinations in patients from the COPD Gene cohort. J Cardiovasc Comput Tomogr. 2011;5: 113–118. doi: 10.1016/j.jcct.2010.11.002 21167806 PMC3075464

[15] Kim SM, Chung MJ, Lee KS, Choe YH, Yi CA, Choe B-K. Coronary Calcium Screening Using Low-Dose Lung Cancer Screening: Effectiveness of MDCT with Retrospective Reconstruction C a r d i a c I m ag i ng • O r ig i n a l R e s e a rc h. 2008 [cited 19 Apr 2020]. doi: 10.2214/AJR.07.2979 18356437

[16] Wang L, Cheng X, Dong Q, Zhou C, Wang Y, Song B, et al. The characteristics of laboratory tests at admission and the risk factors for adverse clinical outcomes of severe and critical COVID-19 patients. BMC Infect Dis. 2021;21: 1–8. doi: 10.1186/s12879-020-05706-z 33879073 PMC8057654

[17] Leulseged TW, Hassen IS, Ayele BT, Tsegay YG, Abebe DS, Edo MG, et al. Laboratory biomarkers of covid-19 disease severity and outcome: Findings from a developing country. PLoS One. 2021;16: e0246087. doi: 10.1371/journal.pone.0246087 33720944 PMC7959358

[18] Keddie S, Ziff O, Chou MKL, Taylor RL, Heslegrave A, Garr E, et al. Laboratory biomarkers associated with COVID-19 severity and management. Clin Immunol. 2020;221. doi: 10.1016/j.clim.2020.108614 33153974 PMC7581344

[19] Jose Antunez Muiños P, López Otero D, Amat-Santos IJ, López País J, Aparisi A, Cacho Antonio CE, et al. The COVID-19 lab score: an accurate dynamic tool to predict in-hospital outcomes in COVID-19 patients. Sci Reports |. 123AD;11: 9361. doi: 10.1038/s41598-021-88679-6 33931677 PMC8087839

[20] Francone M, Iafrate F, Masci GM, Coco S, Cilia F, Manganaro L, et al. Chest CT score in COVID-19 patients: correlation with disease severity and short-term prognosis. Eur Radiol. 2020;30: 6808–6817. doi: 10.1007/s00330-020-07033-y 32623505 PMC7334627

[21] A Grammar of Data Manipulation [R package dplyr version 1.0.6]. 2021 [cited 16 Jun 2021]. Available: https://cran.r-project.org/package=dplyr

[22] Nagelkerke NJD. A note on a general definition of the coefficient of determination. Biometrika. 1991. doi: 10.1093/biomet/78.3.691

[23] John F, Sanford W. An R Companion to Applied Regression Third Edition. SAGE Publications, Inc. 2019.

[24] Midi H, Sarkar SK, Rana S. Collinearity diagnostics of binary logistic regression model. J Interdiscip Math. 2010;13: 253–267. doi: 10.1080/09720502.2010.10700699

[25] Faul F, Erdfelder E, Lang AG, Buchner A. G*Power 3: A flexible statistical power analysis program for the social, behavioral, and biomedical sciences. Behavior Research Methods. Psychonomic Society Inc.; 2007. pp. 175–191. doi: 10.3758/bf03193146 17695343

[26] Revelle W. psych: Procedures for Personality and Psychological Research. In: https://CRAN.R-project.org/package=psych. 2020.

[27] Koo TK, Li MY. A Guideline of Selecting and Reporting Intraclass Correlation Coefficients for Reliability Research. J Chiropr Med. 2016;15: 155–163. doi: 10.1016/j.jcm.2016.02.012 27330520 PMC4913118

[28] Lessmann N, Van Ginneken B, Zreik M, De Jong PA, De Vos BD, Viergever MA, et al. Automatic Calcium Scoring in Low-Dose Chest CT Using Deep Neural Networks with Dilated Convolutions. IEEE Trans Med Imaging. 2018;37: 615–625. doi: 10.1109/TMI.2017.2769839 29408789

[29] Nai Fovino L, Cademartiri F, Tarantini G. Subclinical coronary artery disease in COVID-19 patients. Eur Hear J—Cardiovasc Imaging. 2020 [cited 10 Aug 2020]. doi: 10.1093/ehjci/jeaa202 32671381 PMC7454480

[30] Dillinger JG, Benmessaoud FA, Pezel T, Voicu S, Sideris G, Chergui N, et al. Coronary Artery Calcification and Complications in Patients With COVID-19. JACC Cardiovasc Imaging. 2020;13: 2468–2470. doi: 10.1016/j.jcmg.2020.07.004 33153535 PMC7605736

[31] Giannini F, Toselli M, Palmisano A, Cereda A, Vignale D, Leone R, et al. Coronary and total thoracic calcium scores predict mortality and provides pathophysiologic insights in COVID-19 patients. J Cardiovasc Comput Tomogr. 2021 [cited 17 Jun 2021]. doi: 10.1016/j.jcct.2021.03.003 33744175 PMC7946543

[32] Ritter M, Ott DVM, Paul F, Haynes JD, Ritter K. COVID-19: a simple statistical model for predicting intensive care unit load in exponential phases of the disease. Sci Rep. 2021;11: 5018. doi: 10.1038/s41598-021-83853-2 33658593 PMC7930200

[33] Christensen JL, Sharma E, Gorvitovskaia AY, Watts JP, Assali M, Neverson J, et al. Impact of slice thickness on the predictive value of lung cancer screening computed tomography in the evaluation of coronary artery calcification. J Am Heart Assoc. 2019;8. doi: 10.1161/JAHA.118.010110 30620261 PMC6405734

[34] Deprez FC, Vlassenbroek A, Ghaye B, Raaijmakers R, Coche E. Controversies about effects of low-kilovoltage MDCT acquisition on Agatston calcium scoring. J Cardiovasc Comput Tomogr. 2013;7: 58–61. doi: 10.1016/j.jcct.2012.11.006 23333185

