# Supplementary material for: Coronary artery calcification on low-dose chest CT is an early predictor of severe progression of COVID-19—A multi-center, multi-vendor study
Source: PLoS One. 2021 Jul 21;16(7):e0255045. doi: 10.1371/journal.pone.0255045 (PMC8294495; doi:10.1371/journal.pone.0255045)
Supplement: S1 File — (DOCX) [file pone.0255045.s002.docx]

**Statistical power analysis in G*Power**

To ensure adequate statistical power of our methodology, we performed a post-hoc analysis using the open source software *G*Power* (Figure 1)[1].


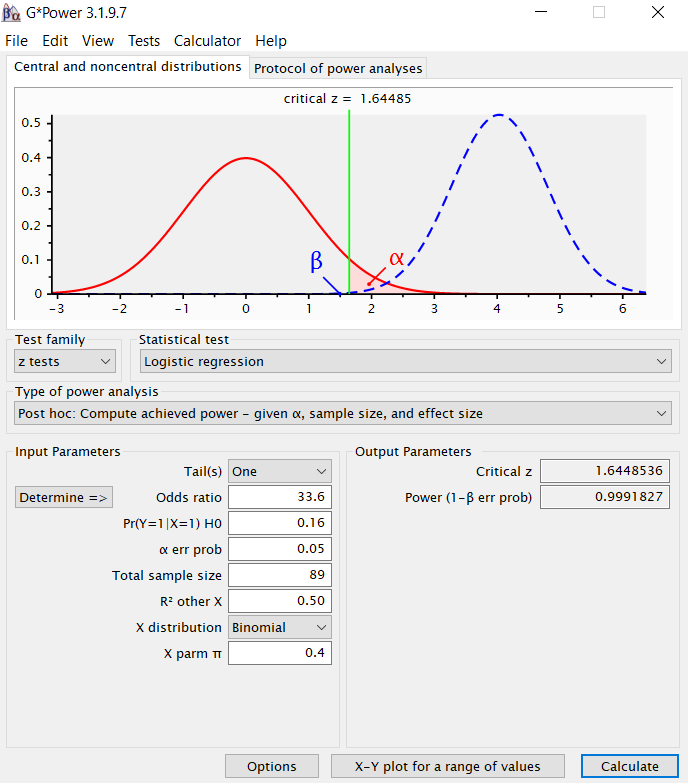


**Figure 1: *G*Power* screenshot for post-hoc computation of statistical power of a one-tailed, multivariate logistic regression.**

Input parameters were defined post-hoc according to our study population:
1. Odds ratio = 33.6
2. Pr(Y=1|X=1) H0 = 0.16 (probability that the primary endpoint was achieved, given the Agatston score was <0)
3. Alpha error (level of statistical significance) = 0.05
4. Sample size n = 89 patients
5. R² of control variables, excluding the main independent variable = 0.50
6. Distribution of the main independent variable: Binomial (Agatston score >0 / <0)
7. X parm π (the portion of patients with Agatston score >0) = 0.40 (36/89 patients)

The post hoc analysis yielded a statistical power of >0.99, which exceeds the typically desired power level of 0.80.

**References**

1. Faul F, Erdfelder E, Lang AG, Buchner A. G*Power 3: A flexible statistical power analysis program for the social, behavioral, and biomedical sciences. Behavior Research Methods. Psychonomic Society Inc.; 2007. pp. 175–191. doi:10.3758/BF03193146
