# Supplementary material for: Coronary artery calcification on low-dose chest CT is an early predictor of severe progression of COVID-19—A multi-center, multi-vendor study
Source: PLoS One. 2021 Jul 21;16(7):e0255045. doi: 10.1371/journal.pone.0255045 (PMC8294495; doi:10.1371/journal.pone.0255045)
Supplement: S2 File — (DOCX) [file pone.0255045.s003.docx]

**Intra- and inter-observer variability analysis**

Intra- and inter-observer variability were reported by the intraclass correlation coefficient (ICC), using the R library *psych*: Procedures for Psychological, Psychometric, and Personality Research [1,2].

Intra- and inter-observer variability were obtained by a two-way mixed-effects model (ICC3) and a two-way random-effects model (ICC2) for single ratings, respectively. Intra-observer variability analysis of Agatston score measurements yielded an ICC3=0.96, while inter-observer variability was represented by an ICC2=0.95 (Table 1). For lung involvement scoring, intra-observer variability yielded an ICC3=0.95, while inter-observer variability yielded an ICC2=0.93 (Table 2).

Both intra- and inter-observer reliability were excellent (ICC >0.90).

| Patient | Agatston score Reader 1, Reading 1 | Agatston score Reader 1, Reading 2 | Agatston score Reader 2 |
| --- | --- | --- | --- |
| 1 | 0 | 0 | 0 |
| 2 | 0 | 0 | 0 |
| 3 | 0 | 0 | 0 |
| 4 | 0 | 0 | 0 |
| 5 | 0 | 0 | 0 |
| 6 | 96.0 | 92.4 | 92.4 |
| 7 | 389.5 | 389.5 | 388.7 |
| 8 | 0 | 0 | 0 |
| 9 | 0 | 0 | 1.3 |
| 10 | 0 | 0 | 0 |
| 11 | 0 | 0 | 0 |
| 12 | 879.3 | 982.5 | 982.5 |
| 13 | 1951.8 | 2620.4 | 2752.7 |
| 14 | 127.0 | 132.1 | 64.0 |
| 15 | 19.2 | 41.8 | 18.3 |
| 16 | 0 | 0 | 0 |

**Table 1. Agatston score measurements for assessment of intra- and inter-observer variability.**

| Patient | Lung involvement score Reader 1, Reading 1 | Lung involvement score Reader 1, Reading 2 | Lung involvement score Reader 2 |
| --- | --- | --- | --- |
| 1 | 17 | 14 | 14 |
| 2 | 7 | 9 | 8 |
| 3 | 6 | 6 | 5 |
| 4 | 5 | 6 | 7 |
| 5 | 15 | 16 | 16 |
| 6 | 1 | 1 | 1 |
| 7 | 22 | 21 | 22 |
| 8 | 8 | 11 | 9 |
| 9 | 16 | 18 | 15 |
| 10 | 16 | 19 | 18 |
| 11 | 14 | 12 | 16 |
| 12 | 14 | 14 | 16 |
| 13 | 13 | 15 | 17 |
| 14 | 10 | 12 | 11 |
| 15 | 9 | 10 | 10 |
| 16 | 12 | 12 | 16 |

**Table 2. Lung involvement scores for assessment of intra- and inter-observer variability.**

References

1. Revelle W. psych: Procedures for Personality and Psychological Research. In: https://CRAN.R-project.org/package=psych. 2020.

2. Koo TK, Li MY. A Guideline of Selecting and Reporting Intraclass Correlation Coefficients for Reliability Research. J Chiropr Med. 2016;15: 155–163. doi:10.1016/j.jcm.2016.02.012
